# Genetic predisposition for negative affect predicts mental health burden during the COVID-19 pandemic

**DOI:** 10.1007/s00406-024-01795-y

**Published:** 2024-04-08

**Authors:** Alicia M. Schowe, Malvika Godara, Darina Czamara, Mazda Adli, Tania Singer, Elisabeth B. Binder

**Affiliations:** 1 Department of Genes and Environment, Max Planck Institute of Psychiatry, Munich, Germany; 2 Graduate School of Systemic Neuroscience, Ludwig Maximilian University, Munich, Germany; 3 Social Neuroscience Lab, Max Planck Society, 10557 Berlin, Germany; 4 Department of Psychiatry and Neurosciences, CCM, Charité-Universitätsmedizin Berlin, Berlin, Germany; 5 Center for Psychiatry, Psychotherapy and Psychosomatic Medicine, Fliedner Klinik Berlin, Berlin, Germany

**Keywords:** Polygenic risk scores, Mental health, Vulnerability, Resilience, COVID-19, Pandemic

## Abstract

The coronavirus disease 2019 (COVID-19) pandemic was accompanied by an increase in mental health challenges including depression, stress, loneliness, and anxiety. Common genetic variants can contribute to the risk for psychiatric disorders and may present a risk factor in times of crises. However, it is unclear to what extent polygenic risk played a role in the mental health response to the COVID-19 pandemic. In this study, we investigate whether polygenic scores (PGSs) for mental health-related traits can distinguish between four resilience-vulnerability trajectories identified during the COVID-19 pandemic and associated lockdowns in 2020/21. We used multinomial regression in a genotyped subsample (*n* = 1316) of the CovSocial project. The most resilient trajectory characterized by the lowest mental health burden and the highest recovery rates served as the reference group. Compared to this most resilient trajectory, a higher value on the PGS for the well-being spectrum decreased the odds for individuals to be in one of the more vulnerable trajectories (adjusted *R*-square = 0.3%). Conversely, a higher value on the PGS for neuroticism increased the odds for individuals to be in one of the more vulnerable trajectories (adjusted *R*-square = 0.2%). Latent change in mental health burden extracted from the resilience-vulnerability trajectories was not associated with any PGS. Although our findings support an influence of PGS on mental health during COVID-19, the small added explained variance suggests limited utility of such genetic markers for the identification of vulnerable individuals in the general population.

**Supplementary Information:**

The online version contains supplementary material available at 10.1007/s00406-024-01795-y.

## Introduction

The coronavirus disease 2019 (COVID-19) pandemic and societal measures imposed to contain its spread have been accompanied by severe socio-economic and health-related burdens worldwide [1–3]. A large body of literature on the pandemic’s initial effects, declared a global pandemic accompanied by recommendations for social isolation in March 2020 [4], shows an increase in population-level anxiety, depression, psychological distress, suicidal ideation, and loneliness [5–11], with most severe and enduring impairments in individuals of younger age, female sex, and lower socio-economic status (SES) [7, 10, 12–22]. The majority of these studies has focused on identifying the role of demographic characteristics and socioeconomic living conditions as risk factors for mental health challenges. However, individual differences in mental health impairment may also be influenced by genetic predisposition. Genome-wide association studies (GWAS) have identified a wide range of common genetic variants and biological pathways contributing to an increased risk of major depressive [23] and anxiety disorders [24], loneliness [25], and suicidal ideation [26]. Moreover, some studies suggest that the risk of experiencing adverse exposures [27–29] or the impact of an adverse exposure on mental health can differ depending on the genetic predisposition for psychiatric disorders such as major depressive disorder (MDD) [30–33]. In the context of the COVID-19 pandemic, polygenic risk for psychiatric disorders has been associated with increased risk of any COVID-19 and severe COVID-19 infection [34], greater COVID-19-related threat and crisis perception [35], and greater physical and mental health impairments [35, 36], suggesting polygenic predisposition for psychiatric disorders as a risk factor for worse health outcomes in times of such collective crises.

However, not all studies support the role of polygenic risk in the mental health response to the COVID-19 pandemic [37]. Polygenic risk on the individual level can be assessed using polygenic scores (PGSs) which are computed as the weighted sum of an individual’s genetic risk variants previously associated with a given trait of interest in genome-wide association analyses [38]. PGSs thus represent an individual’s additive genetic predisposition for a given common trait in a single score. Previous studies addressing mental health during Covid-19 differ in the selected PGS, COVID-19-related timing, and mental health outcome. When adjusting for pre-pandemic baseline symptoms, Taylor et al. [37], for example, show that the PGSs for MDD, anxiety and loneliness were not associated with the corresponding phenotype observed in June-July and November–December 2020. Warmerdam et al. [35], on the other hand, report a positive effect of the life satisfaction PGS on perceived quality of life over a 10-month period along with significant associations of several PGS including MDD and educational attainment with mental health at baseline in March 2020. Taking genetic correlations between PGS into account, Ahrens et al. [36] found a significant positive association between a pleiotropic neurodevelopmental genetic factor (consisting of two PGSs), but no other genetic factor, and a more acute mental dysfunction trajectory during the first COVID-19 lockdown. Pleiotropic mechanisms between psychiatric disorders have been demonstrated in a growing number of studies [39–43]. Lee et al., for instance, identified three correlated genetic factors, which together explained 51% of the genetic variation in eight combined neuropsychiatric disorders [43]. Moreover, Grotzinger et al. [39] found four genetic factors (neurodevelopmental, compulsive, psychotic, and internalizing) with, in part, distinct biobehavioral correlates. PGS based on these broad genetic factors may better represent a genetic vulnerability to the cumulative burden of complex stressors such as the COVID-19 pandemic and may account for more phenotypic variation in mental health outcomes compared to individual PGS. Yet, most previous studies have focused on individual PGSs.

The present study was embedded in the CovSocial project, which is a longitudinal study of the effects of COVID-19 pandemic in 2020 and 2021 on various biopsychological outcomes in *n* = 3522 individual living in Berlin [44]. Repeated assessments of mental burdens took place over 7 measurement time points covering two lock-downs: T1 (before the lockdown in January 2020), T2 (during the first lockdown from mid-March to mid-April 2020), T3 (in June 2020 when restrictions were eased), T4 (November 2020), T5 (December 2020), T6 (January 2021), and T7 (mid-March to mid-April 2021, see Online Resource 2, Fig. 1). Building on prior frameworks, the recent Wither or Thrive Model of Resilience[45] conceptualized resilience during the COVID-19 pandemic as a dynamic process evolving over time in response to recurring stressors, manifesting as distinct resilience-vulnerability trajectories. To assess differential resilience-vulnerability trajectories in the CovSocial project[13] first a latent factor summarizing many different vulnerability indicators (e.g., depression, loneliness, stress, covid burden, etc.) and resilience indicators (e.g., optimism, coping, resistance) was built (see Online Resource S2, Fig. 2). Then, using growth mixture models, study participants were categorized into four resilience-vulnerability trajectories covering the entire time span from 2020 to 2021 including two lock-downs: a most vulnerable group (12.8%) with greatest mental health challenges reflected not only in more mental health challenges at baseline but also slower recovery rates and stronger pandemic fatigue effects during lock-downs, a more vulnerable group (16.7%), a more resilient group (46%), and a most resilient group (24.5%) with least mental health challenges at baseline, recovery to baseline after the first lockdown and lowest pandemic fatigue effect during the long second lock-down [13].

In the present investigation, we test whether individual PGS and PGS derived from latent phenotypes are associated with differences in these resilience-vulnerability trajectories and whether PGS derived from latent phenotypes explain greater phenotypic variation in mental health burden compared to individual PGS. Moreover, we explore the associations of the PGS with baseline and baseline independent change scores of latent mental health burden during the first and second COVID-19 lockdown in Germany.

## Methods

### Sample

Recruitment and inclusion criteria of the multi-phase CovSocial project are described in Godara et al. [13] and the Online Resource S1. This study is based on a genotyped subsample of the first phase of the CovSocial project which focuses on the impact of pandemic-related lockdowns in Germany on various biopsychological domains including mental health burden and vulnerability (*n* = 3522, age 18–65 years, 65.11% female). Characteristics of the analytic sample of this study (*n* = 1316) are reported in Table 1. Compared to the full Phase 1 CovSocial sample, participants in the analytic sample were significantly older, more often in the above-average income group, more often diagnosed with a lifetime mental disorder, less often in the most vulnerable group, and scored on average lower in latent vulnerability at T4 (see Online Resource S3, Table 1).

**Table 1:** Sample characteristics

Characteristic	Overall, *N* = 1316	Most Vulnerable, *N* = 128^*1*^	More vulnerable *N* = 296	More resilient, *N* = 575	Most resilient, *N* = 317	*p*^a^
Age [mean (SD)]	45.31 (12.45)	42.96 (12.58)	43.26 (11.98)	44.98 (12.71)	48.75 (11.64)	** < 0.001**
Female [ (%)]	882 (67%)	91 (71%)	236 (80%)	400 (70%)	155 (49%)	** < 0.001**
Income < Berlin average [*n* (%)]	426 (32%)	53 (41%)	129 (44%)	185 (32%)	59 (19%)	** < 0.001**
Lifetime mental disorder [*n* (%)]	398 (30%)	77 (60%)	128 (43%)	165 (29%)	28 (8.8%)	** < 0.001**
Years in education [mean (SD)]	17.23 (3.76)	17.50 (3.66)	17.71 (3.32)	17.02 (3.78)	17.04 (4.12)	**0.024**
Education missing [*n*]	2	0	1	1	0	
Latent vulnerability T1 [mean (SD)]	− 0.05 (0.92)	1.08 (1.11)	0.19 (0.99)	− 0.01 (0.67)	− 0.80 (0.43)	** < 0.001**
Latent Vulnerability T4 [mean (SD)]	0.59 (1.26)	2.60 (0.71)	1.12 (1.12)	0.47 (0.81)	− 0.67 (0.62)	** < 0.001**
T4 missing [*n*]	512 (38.9%)	47 (36.7%)	87 (29.4%)	245 (44.2%)	133 (42.0%)	
LCS1 [mean (SD)]	0.71 (0.96)	1.51 (1.21)	0.87 (1.20)	0.67 (0.84)	0.33 (0.42)	** < 0.001**
LCS2 [mean (SD)]	− 0.53 (0.76)	− 0.74 (1.09)	− 0.58 (1.07)	− 0.57 (0.63)	− 0.33 (0.29)	** < 0.001**
Slope T4–T7 [mean (SD)]	0.05 (0.11)	0.06 (0.07)	0.07 (0.18)	0.06 (0.07)	0.03 (0.05)	**0.001**
Slope missing [*n*]	512	47	87	245	133	

*p*-values marked in bold indicate significance at *p* < 0.05

*LCS1* latent change score from T1 to T2, *LCS2* latent change score from T2 to T3, *SD* standard deviation

^a^Kruskal-Wallis rank sum test for differences in continuous and Pearson’s Chi-squared test for differences in categorical variables between the four resilience-vulnerability trajectories

The study was approved by the Ethics Committee of Charité—Universitätsmedizin Berlin (#EA4/172/20 and #EA1/345/20) and was conducted in accordance with the Declaration of Helsinki. All participants provided written informed consent prior to participation.

### Longitudinal measures and study design

**Fig. 1:**
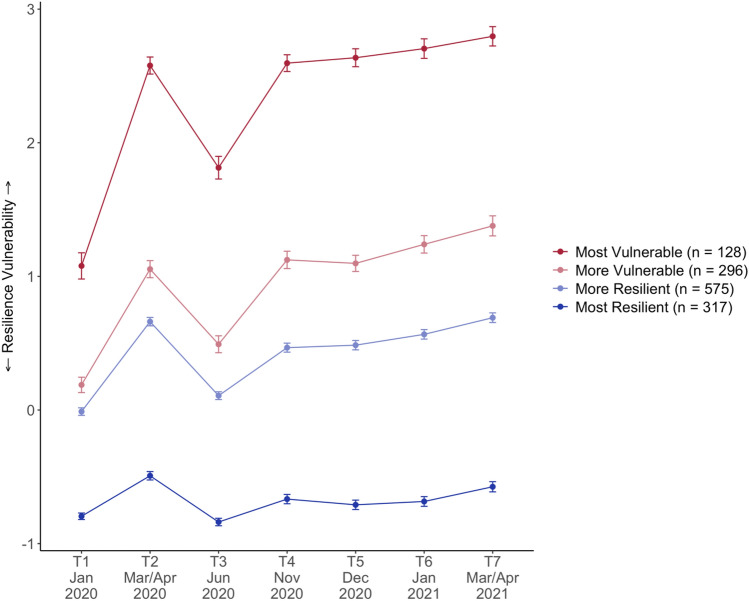
Resilience-vulnerability trajectories in the analytic sample

Mental health burden and vulnerability measures consisted of both validated scales and self-generated questions. Stress perception, self-efficacy beliefs, and depressive and anxiety symptoms were assessed using the Perceived Stress Scale (PSS-4 [46]), the General Self-Efficacy Short Scale (ASKU [47]), the Patient Health Questionnaire-2 (PHQ-2 [48]), and the Generalized Anxiety Disorder Scale (GAD-2 [49]), respectively. Self-generated questions were developed for capturing aspects of resilience vulnerability that are specific to the given pandemic and its dynamic nature (e.g., pandemic-related burdens, loneliness, life satisfaction). A full list of these questions and detailed study design can be found in the Online Resource S2. As shown in Online Resource S2, Fig. 1, data were collected with repeated online surveys at seven timepoints: T1 (before the lockdown in January 2020), T2 (during the first lockdown from mid-March to mid-April 2020), T3 (in June 2020 when restrictions were eased), T4 (November 2020), T5 (December 2020), T6 (January 2021), and T7 (mid-March to mid-April 2021). The first three timepoints were assessed retrospectively, in three separate blocks of questionnaires, from 11 September 2020 to 7 December 2020. For the last four time points (T4–T7), participants answered the blocks of 11 questionnaires at the end of each month and rated their feelings, perceptions, and behavior for the last 4 weeks.

As previously described [13] and illustrated in Online Resource 2, Fig. 2, at each of the seven timepoints a latent resilience-vulnerability factor was derived from all measures. Change in the seven extracted factor scores over time was then modelled using latent change score (LCS) and growth curve mixture modelling techniques in Mplus. Changes in resilience-vulnerability from T1 to T2 (acute lockdown effect) and from T2 to T3 (effect of re-opening) were assessed by modeling the differences between the respective time points as latent variables and are referred to as LCS1 (T1–T2) and LCS2 (T2–T3). A linear growth function was specified for T4–T7 with the latent intercept factor positioned at T4. Change from T4 to T7 is referred to as Slope T4–T7 in the following. To identify subpopulations with different mean growth trajectories, mixture analysis was applied to the growth model, resulting in four groups: the most vulnerable (12.8%) with the highest mental health burden, more vulnerable (16.7%), more resilient (46%), and most resilient group (24.5%) with the lowest mental health burden (see Table 1 for group sizes of the analytic sample used in this study).

### Genotype data

DNA was isolated from saliva samples for *n* = 1535 participants and genome-wide SNP genotyping performed using Illumina Genome Screening arrays (GSA) version 3 according to the manufacturer’s guidelines (Illumina Inc., San Diego, CA). Quality control was performed in Plink 1.9 [50]. SNPs with a minor allele frequency below 1%, a call rate below 98%, or with deviation from Hardy–Weinberg-Equilibrium with a *p* value < 1 × 10^–05^ were excluded. Furthermore, SNPs mapping to multiple locations as well as duplicated variants were removed.

Individuals with a genotype call rate below 98% (*n* = 48) and technical replicates (*n* = 12) were excluded. Any pair of samples with IBD estimates > 0.125 was checked for relatedness and for *n* = 6 confirmed. One sample of each pair was removed. Furthermore, individuals showing discrepancies between phenotypic and genotypic sex (*n* = 4) were removed. To retrieve ancestry-related information, we performed multi-dimensional scaling (MDS) analysis on the IBS matrix of quality-controlled genotypes [51]. Outliers, defined as samples presenting with a position on any of the first ten axes of variation deviating more than four standard deviations from the respective axis’ mean, were iteratively removed until no more outliers were detected (*n* = 55). Afterwards, individuals presenting with heterozygosity values more than four standard deviations away from the mean heterozygosity were also iteratively removed (*n* = 3). The first two MDS components (38.5% variance explained) were extracted and included as covariates in the following analyses to account for population structure.

Quality-controlled genotypes were then phased using SHAPEIT [52] and SNPs imputed using impute v2 [53] using the European 1,000 Genomes build 37 phase 3 sample as reference panel [54]. Imputed SNPs with a low information content metric (< 0.6), significant deviation from HWE (*p* < 10^−05^), or low MAF (< 1%) were excluded. Imputed genotype probabilities were converted into best-guessed genotypes using a threshold of 0.90. After quality control, genotype data were available for 1,407 subjects. Of these, *n* = 1316 samples with complete phenotypic information on resilience-vulnerability class and covariates were included in the following analyses.

### Polygenic scores

Fifteen individual and four PGS derived from latent phenotypes were computed based on publicly available GWAS summary statistics (see Table 2). A PGS for height (based on Yengo et al. [55]) was added as a negative control. PGS were generated using PRS-CS [56] and PLINK 1.9 [50]. PRS-CS was applied to infer posterior mean effects by chromosome for autosomal single nucleotide polymorphisms (SNPs) that overlap between the given discovery genome-wide association study (GWAS) and the European 1000 Genomes linkage disequilibrium (LD) panel [54]. To ensure convergence of the underlying Gibbs sampler algorithm, we set Markov chain Monte Carlo (MCMC) iterations to 10,000 and the first 5000 MCMC iterations as burn-in [57]. For all other PRS-CS parameter the default settings were used. Using the PLINK 1.9 score function, raw PGSs were then calculated for each participant as the average risk allele count weighted by the posterior means effects returned by PRS-CS, and then standardized to cohort mean = 0 and SD = 1 in R [58] (4.1.3 (2022-03-10).

**Table 2:** GWAS summary statistics

Trait	References	URL	*N*
Individual GWAS			
Educational attainment (EA)	Okbay et al. [67]	https://www.ebi.ac.uk/gwas/studies/GCST003676	765,283
Life satisfaction (LiS)	Baselmans et al. [68]	https://www.ebi.ac.uk/gwas/publications/30643256	80,852
Positive affect (PA)	Baselmans et al. [68]	https://www.ebi.ac.uk/gwas/studies/GCST007341	410,603
Loneliness (lone)	Day et al. [25]	https://www.ebi.ac.uk/gwas/studies/GCST006924	487,647
Neuroticism (general NEU)	Hill et al. [60]	https://www.ebi.ac.uk/gwas/studies/GCST007710	270,059
Neuroticism (NEU)	Baselmans et al. [68]	https://www.ebi.ac.uk/gwas/publications/30643256	582,989
Depressive symptoms (DEP)	Baselmans et al. [68]	https://www.ebi.ac.uk/gwas/publications/30643256	1,295,946
Worry vulnerability (WoV)	Hill et al. [60]	https://www.ebi.ac.uk/gwas/studies/GCST007710	270,059
Anxiety-tension (AnT)	Hill et al. [60]	https://www.ebi.ac.uk/gwas/studies/GCST007710	270,059
Anxiety disorder (ANX)	Otowa et al. [24]	https://figshare.com/articles/dataset/anx2016/14842689	18,186
Major depressive disorder (MDD)	Howard et al. [23]	https://datashare.ed.ac.uk/handle/10283/3203	500,199
Autism spectrum disorder (ASD)	Grove et al. [69]	https://figshare.com/articles/dataset/asd2019/14671989	46,342
Attention-deficit-hyperactivity disorder (ADHD)	Demontis et al. [70]	https://figshare.com/articles/dataset/adhd2019/14671965	55,374
Posttraumatic stress disorder (PTSD)	Nievergelt et al. [71]	https://figshare.com/articles/dataset/ptsd2019/14672133	206,655
Tourrette syndrome (TS)	Yu et al. [72]	https://figshare.com/articles/dataset/ts2019/14672232	14,307
Latent GWAS			
Subjective well-being spectrum (WBS): NEU, depressiveness, PA, LiS	Baselmans et al. [68]	https://www.ebi.ac.uk/gwas/studies/GCST007341	2,311,184
Internalizing factor (INT): MDD, ANX, PTSD	Grotzinger et al. [73]	https://drive.google.com/drive/folders/1qX3IF0Z_gObcNYmsdIG7bVRJ67nnwZUn	NA
Neurodevelopmental factor (ND): ADHD, ASD, MDD, PTSD, TS	Grotzinger et al. [73]	https://drive.google.com/drive/folders/1qX3IF0Z_gObcNYmsdIG7bVRJ67nnwZUn	NA
General psychopathology (general P)	Grotzinger et al. [73]	https://drive.google.com/drive/folders/1qX3IF0Z_gObcNYmsdIG7bVRJ67nnwZUn	NA
Negative control			
Height (H)	Yengo et al. [55]	https://www.ebi.ac.uk/gwas/studies/GCST006900	693,529

*NA* not available

### Covariates

As shown by Godara et al. [13], and also valid for this subsample, see Table 1, younger age, female sex, and lower than average household income, were associated with more vulnerable resilience-vulnerability trajectories, and thus controlled for in our analyses. Self-reported history of lifetime mental disorder was not included as a covariate because we theorize that both a diagnosis and a more vulnerable trajectory would be an outcome of polygenic risk instead of a confounding factor.

### Statistical analyses

All statistical analyses were conducted in R [58] (version 4.1.3, 2022-03-10). Statistical significance was tested at a 5% level and multiple testing accounted for using the false discovery rate (FDR) for the 20 (correlated) predictors. The analysis code is publicly available at https://github.com/aschowe/CovSocial_PGS_Analysis_2023.git.

#### PGS validity and specificity

For PGS with available corresponding phenotype (e.g., life satisfaction PGS and trait life satisfaction item), we assessed validity using linear regression with the given phenotype as dependent and PGS as independent variable, controlling for the first two ancestral components. For each phenotype, we repeated these analyses using all computed PGS individually and ranked the explained phenotypic variance (in the form of adjusted R squared) to assess PGS specificity.

#### Polygenic scores and mental health burden during the COVID-19 lockdowns

To identify PGS that are associated with the resilience-vulnerability trajectories, we first tested for mean differences using one-way analysis of variance (ANOVA). We then further examined which specific resilience-vulnerability trajectory the identified PGS can distinguish using multinomial regression (nnet R-package (version 7.3-17) [59]). In this analysis, resilience-vulnerability trajectory was used as a dependent and PGS separately as an independent variable, controlling for the two ancestry principal components, age, sex, and income. The most resilient resilience-vulnerability trajectory served as the reference. To obtain an estimate of the added variance explained by PGS, we compared the adjusted R-squared between the model including the PGS to a model including only the phenotypic covariates. We repeated these analyses using linear regression (stats R-package, version 4.1.3 [58] with latent mental health burden at T1, T4, and latent change slopes between T1–T2, T2–T3, and T4–T7 (see Fig. 1) as the dependent variable to disentangle the association of PGS with latent baseline and change scores.

Average latent resilience-vulnerability factor scores are plotted for the analytic sample at each of the seven-time points for the four identified resilience-vulnerability trajectories from most vulnerable (top red line) to most resilient (bottom purple line). Further information on the resilience-vulnerability trajectories in the full CovSocial sample are provided in Godara et al. [13]. Error bars indicate standard error.

## Results

Table 1 shows the demographic background of the analytic sample. Previously reported demographic differences among the four latent resilience-vulnerability trajectories were also seen in our subsample and thus accounted for using covariates in all analyses (see methods).

Correlation coefficients between all PGS can be found in the Online Resource S3, Table 2 and ranged from − 0.83 to − 0.01 and 0.01–0.89, in line with previous reports [39, 40, 60, 61]. We tested 15 individual PGS and 4 PGS derived from latent phenotypes, listed in Table 2. These included individual PGS derived from GWAS for psychiatric disorders, personality traits but also loneliness and well-being. The PGS derived from latent phenotypes were derived from GWAS of subjective well-being as well as a GWAS describing a general psychopathology, neurodevelopmental and internalizing factor.

We first tested how much variance the individual as well as PGS derived from latent phenotypes explained for the different phenotypes and observed explained variances from 0 to 5.6% in trait and mean symptom outcomes across all measurement occasions (see Online Resource, Figs. 1–6). Except for the anxiety disorder PGS (*b* = 0.33, adj. *R*-square = 0.001, *p* = 0.25), each individual PGS was significantly associated with their respective phenotype (i.e., depressiveness PGS—mean depressive symptoms (*b* = 0.07, adj. *R*-square = 0.016, *p* < 0.001), life satisfaction PGS—trait life satisfaction (*b* = 0.45, adj. *R*-square = 0.006, *p* = 0.007), loneliness PGS—trait loneliness (*b* = 0.07, adj. *R*-square = 0.009, *p* < 0.001,), anxiety-tension PGS—trait anxiety (*b* = 0.90, adj. *R*-square = 0.007, *p* = 0.002), neuroticism PGS—trait neuroticism (*b* = 1.32, adj. *R*-square = 0.023, *p* < 0.001,), general factor neuroticism PGS—trait neuroticism (b = 1.27, adj. *R*-square = 0.021, *p* < 0.001), educational attainment PGS—educational years (*b* = 0.88, adj. *R*-square = 0.056, *p* < 0.001,). The well-being spectrum PGS explained the largest amount of variance compared to all other PGS in mean depressive symptoms (*b* = − 0.09, adj. *R*-square = 0.027, *p* < 0.001), trait anxiety (*b* = − 2.09, adj. *R*-square = 0.038, *p* < 0.001), trait neuroticism (*b* = − 1.55, adj. *R*-square = 0.031, *p* < 0.001), and trait life satisfaction (*b* = 0.85, adj. *R*-square = 0.021, *p* < 0.001).

**Fig. 2:**
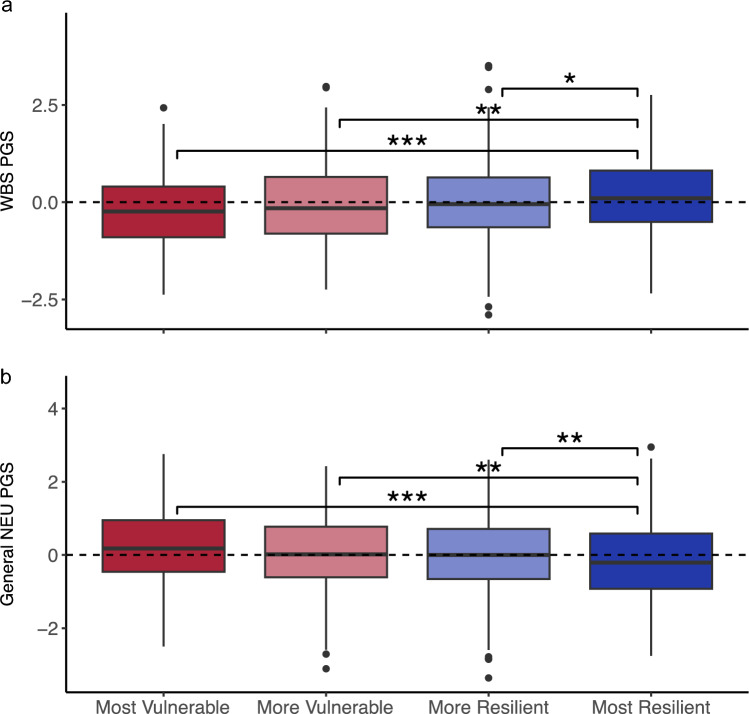
Association between well-being PGS and resilience-vulnerability trajectory. Boxplots of the well-being PGS (**a**) and neuroticism PGS (**b**) are shown by resilience-vulnerability trajectory from the most vulnerable (left) to the most resilient (right) trajectory. Individuals with a higher value on the well-being PGS or a lower value on the General neuroticism PGS were more likely to be in the most resilient group compared to all other groups. *PGS* Polygenic Score, *WBS* well-being spectrum, *NEU* neuroticism. * < 0.05, **p < 0.01, ***p < 0.001

We next tested the associations of the PGS levels with the four resilience-vulnerability trajectories. We found that of the 20 individual PGS and PGS derived from latent phenotypes (see Table 3), the PGS for well-being (*F*(3) = 6.76, *p* = 0.0002), general neuroticism factor (*F*(3) = 5.21, *p* = 0.001), neuroticism (*F*(3) = 4.76, *p* = 0.003), MDD (*F*(3) = 3.39, *p* = 0.017), Tourette syndrome (*F*(3) = 3.35, *p* = 0.018), general psychopathy factors (*F*(3) = 3.23, *p* = 0.022), depressiveness (*F*(3) = 3.19, *p* = 0.023), and internalizing (*F*(3) = 2.72, *p* = 0.043) significantly differed between the four resilience-vulnerability trajectories. However, only associations with the well-being (FDR = 0.003) and general neuroticism factor PGS (*p* adj. = 0.02) remain significant after multiple testing correction (see Table 3 for an overview of all PGS results). Following up on these mean differences in PGS, we observe that individuals with lower well-being were less likely to be in the most vulnerable (OR = 0.63, *p* < 0.001), more vulnerable (OR = 0.78, *p* = 0.004), and more resilient (OR = 0.86, *p* = 0.039) trajectory compared to the most resilient group (adj. *R*-square = 0.3%, see Fig. 2a). Conversely, individuals with a higher factor neuroticism PGS were significantly more likely to be in the most vulnerable (OR = 1.48, *p* < 0.001), more vulnerable (OR = 1.29, *p* = 0.003), and more resilient (OR = 1.21, *p* = 0.009) trajectory compared to the most resilient group (adjusted *R*-square = 0.2%, see Fig. 2b). Both the well-being (added adj. *R*-square = 0.21%, *p* = 0.008) and the general neuroticism PGS (added adj. *R*-square = 0.2%, *p* = 0.023) significantly explained additional variance compared to the covariate-only model. The height PGS as a negative control did not differ between the resilience-vulnerability trajectories (see Tables 3 and 4).

**Table 3:** One-way ANOVA of PGS and resilience-vulnerability trajectory

PGS	PGS mean by resilience-vulnerability trajectory				*df*	*F*	*p*	*p*-adj
	Most vulnerable (*n* = 128)	More vulnerable (*n* = 296)	More resilient (*n* = 575)	Most resilient (*n* = 317)				
WBS	− 0.264	− 0.076	0.023	0.17	3	6.762	0.0002	**0.003**
General NEU	0.188	0.063	− 0.001	− 0.176	3	5.213	0.001	**0.02**
NEU	− 0.809	− 0.941	− 1	− 1.163	3	4.757	0.003	0.06
MDD	0.09	0.111	− 0.054	− 0.115	3	3.395	0.017	0.34
TS	0.153	− 0.141	0.032	0.042	3	3.35	0.018	0.36
General P	0.087	0.118	− 0.032	− 0.112	3	3.232	00.022	0.44
DEP	1.156	1.058	0.977	0.869	3	3.196	0.023	0.46
INT	0.126	0.076	− 0.038	− 0.11	3	2.723	0.043	0.86
AnT	0.068	0.048	0.034	− 0.126	3	2.319	0.074	1
Lone	0.188	0.015	− 0.045	− 0.052	3	2.173	0.089	1
PA	− 0.174	− 0.033	0.012	0.069	3	1.949	0.12	1
WoV	0.148	0.038	− 0.008	− 0.084	3	1.825	0.141	1
ASD	− 0.04	0.112	− 0.02	− 0.067	3	1.818	0.142	1
ANX	− 0.065	− 0.069	0.054	− 0.071	3	1.626	0.182	1
EA	− 0.059	0.085	− 0.037	0.026	3	1.209	0.305	1
H	0.054	0.059	− 0.022	− 0.025	3	0.627	0.598	1
PTSD	0.075	− 0.053	− 0.009	0.011	3	0.533	0.66	1
LiS	0.027	0.003	− 0.017	0.007	3	0.088	0.966	1
ADHD	− 0.015	− 0.005	0.005	0.019	3	0.049	0.985	1
ND	− 0.016	0.001	− 0.001	0.017	3	0.038	0.99	1

*p*-values marked in bold indicate significance at *p* < 0.05 after multiple testing correction

PGS mean comparisons between the four resilience-vulnerability trajectories using one-way analysis of variance (ANOVA)

*PGS* polygenic score, *p-adj* false discovery rate (FDR) corrected *p* value for 20 predictors, *MDD* major depressive disorder, *PTSD* post-traumatic stress disorder, *ANX* anxiety disorder, *ADHD* attention deficit hyperactivity disorder, *ASD* autism spectrum disorder, *NEU* neuroticism, *General NEU* neuroticism factor, *AnT* Anxiety Tension, *WoV* Worry-Vulnerability, *WBS* well-being spectrum, *LiS* Life Satisfaction, *Lone* Loneliness, EA educational attainment, *PA* positive affect, *TS* Tourette Syndrome, *INT* internalizing disorders, *ND* neurodevelopmental disorders, *General P* general psychopathology, *DEP* Depressiveness, *H* height

**Table 4:** Association between the PGS and resilience-vulnerability class

PGS	Most vulnerable multinomial odds ratio (95% CI)	More vulnerable multinomial odds ratio (95% CI)	More resilient multinomial odds ratio (95% CI)	Adj. *R*^2^	Added adj. *R*^2^	LRT	Chi^2^ *p*
WBS	0.63*** (0.51, 0.79)	0.78** (0.66, 0.93)	0.86* (0.75, 0.99)	0.035	0.003	22.259	**0.008**
General NEU	1.48*** (1.19, 1.83)	1.29** (1.09, 1.53)	1.21** (1.05, 1.39)	0.034	0.002	19.217	**0.023**
Height PGS	1.11 (0.89, 1.37)	1.09 (0.93, 1.29)	1.01 (0.88, 1.17)	0.031	-0.001	5.499	0.789

*p*-values marked in bold indicate significance at *p* < 0.05

Odds refer to the most resilient group as the reference level. The adjusted *R* squared refers to the full model including covariates. The added adjusted *R*-square and Chi-square *p* value indicate the additional variances explained of the PGS + covariate vs the covariate-only model

*PGS* polygenic score, *CI* confidence interval, *WBS* well-being spectrum, *General NEU* general neuroticism factor

*p < 0.05

**p < 0.01

***p < 0.001

We then also investigated associations at the level of individual latent resilience-vulnerability scores that define the latent resilience-vulnerability trajectories. At T1 and T4, higher latent resilience-vulnerability factor score (indicating greater vulnerability characterized by higher mental health burden) was negatively associated with the well-being PGS (T1: *b* = − 0.13, *p* < 0.001, added adj *R*-square = 2.1%, T4: *b* = − 0.19, *p* < 0.001, added adj. *R*-square = 2.1%) and positively associated with the general neuroticism PGS (T1: *b* = 0.10 *p* < 0.001, added adj *R*-square = 1.3%; T4: *b* = 0.18, *p* < 0.001; added adj *R*-square = 1.9%). Latent change from T1 to T2, T2–T3, and T4–T7 was not associated with any PGS (see Table 5).

**Table 5:** Associations between genetic factor scores and latent resilience-vulnerability scores

PGS	beta	SE	*t* statistic	*p*	*n*	adj. *R*^2^	added adj. *R*^2^	*F*	Pr (> *F*)
Latent-vulnerability at T1									
WBS	− 0.133	0.024	− 5.446	** < 0.0001**	1316	0.096	0.021	10.737	** < 0.0001**
General neu	0.104	0.025	4.253	**0.0001**	1316	0.088	0.013	6.875	**0.0001**
Height PRS	0.014	0.025	0.55	0.583	1316	0.075	0	0.934	0.423
Latent-vulnerability LCS1									
WBS	− 0.004	0.026	− 0.147	0.883	1316	0.024	− 0.001	0.79	0.499
General NEU	0.015	0.026	0.566	0.572	1316	0.025	0	0.889	0.446
Height PRS	0.039	0.026	1.481	0.139	1316	0.026	0.001	1.515	0.209
Latent-vulnerability LCS2									
WBS	0.003	0.021	0.165	0.869	1316	0.022	− 0.001	0.593	0.620
General NEU	0.0002	0.021	− 0.011	0.991	1316	0.022	− 0.001	0.584	0.626
Height PRS	− 0.021	0.021	− 1.011	0.312	1316	0.023	0	0.925	0.428
Latent-vulnerability at T4									
WBS	− 0.19	0.043	− 4.4	**0.0001**	804	0.082	0.021	7.041	**0.0001**
General NEU	0.18	0.044	4.131	**0.0004**	804	0.08	0.019	6.274	**0.0003**
Height PRS	0.013	0.044	0.298	0.766	804	0.06	− 0.001	0.604	0.613
Latent-vulnerability slope T4–T7									
WBS	− 0.005	0.004	− 1.308	0.191	804	0.001	0.002	1.444	0.229
General NEU	0.005	0.004	1.146	0.252	804	0	0.001	1.312	0.269
Height PRS	0.004	0.004	1.097	0.273	804	0	0.001	1.275	0.282

*p*-values marked in bold indicated significance at *p* < 0.05

Separate linear regression analysis of latent baseline and changes scores as an outcome and each PGS that significantly distinguished between the resilience-vulnerability trajectories as predictor. Height PGS was added as negative control. The adjusted *R* squared refers to the full model including covariates. The added adjusted *R*-square, *F*-test statistic, and *F*-test *p* value refer to the PGS + covariate compared to the covariate-only model

*WBS* well-being spectrum, *General NEU* general neuroticism factors PGS, *PGS* polygenic score, *SE* standard error

## Discussion

The present study investigated whether genetic predisposition for mental health-related traits is associated with a more vulnerable resilience-vulnerability trajectory and higher mental health burden during the COVID-19 pandemic. We find that higher values on the general neuroticism PGS and lower values on the well-being spectrum PGS resulted in an increased likelihood to be assigned to a more vulnerable compared to the most resilient trajectory. The largest amount of variance (with an adj. *R*-square of 0.3%) in the resilience-vulnerability response trajectories as well as in baseline latent resilience-vulnerability was explained by the well-being spectrum PGS (with an adj. *R*-square of 2.1% at T1 and T4). Baseline-independent latent change in from T1 to T2, T2–T3, and T4–T7 was not associated with any PGS.

Our findings support the role of genetic predisposition in risk and resilience to mental health problems during the COVID-19 pandemic. Specifically, we find that PGS for emotional well-being-related traits are associated with a more vulnerable resilience-vulnerability trajectory characterized by a higher baseline, higher acute lockdown response, and less recovery. However, the small added variance explained by PGS consistent with previous studies [62–63] suggests still limited utility for the identification of individuals at risk for mental health impairment beyond socioeconomic predictors in the general population. The well-being PGS slightly outperformed all other PGS in predicting the resilience-vulnerability trajectories and baseline mental health burden, supporting that increased GWAS sample size and advances in multivariate methods can improve predictive performance for complex traits [64]. Yet, the latent general psychopathology, neurodevelopmental and internalizing disorder PGS were not associated with the resilience-vulnerability trajectory after multiple testing correction. The improved performance of PGS derived from latent phenotypes may thus only apply when it matches the outcome of interest well (e.g., here a combination of dimensional resilience and psychological vulnerability questionnaires summarized in Fig. S2 assessed in the general population). Differences in mental health outcomes may also explain why we did not find an effect of the neurodevelopmental PGS opposed to Ahrens et al.[36] who focused on mental dysfunction. Finally, none of the PGS was associated with baseline-independent change. These isolated change slopes may not capture well the higher-order mental health responses represented by the latent resilience-vulnerability profiles including global patterns of baseline and longitudinal change over seven timepoints. Yet, the finding is consistent with Taylor et al.’s [37] report of a lack of association between pre-pandemic controlled mental health and MDD, loneliness, and anxiety. The large majority of GWAS to date identify genetic variants based on main effects on a given cross-sectional trait or case–control status and may thus not be designed to predict change in response to specific stressors. Expanding efforts such as genome-by-trauma exposure association studies [65, 66] in terms of sample size and environmental exposures (e.g., socioeconomic hardship) may be promising approaches to improve the predictive power of PGS in the context of stressful life events in the future.

While the CovSocial study included longitudinal assessments, it is important to note that the first three timepoints were assessed retrospectively so that (lack of) association with acute change in mental health should be interpreted with caution. Bias of these retrospective reports was minimized through priming participants at the beginning of each questionnaire using news specific to the occasion of interest and separate assessment of the retrospective reports across three occasions. Another limitation is the small variability in the isolated latent change scores which restricted the statistical analysis. Moreover, mechanistically this study cannot disentangle whether associations with PGS are due to predisposition to lower mood itself or gene-environment correlation in which genetic predisposition, for example, is associated with an environment characterized by greater exposure to Covid-19-related burdens and is therefore associated with a more vulnerable trajectory. Nevertheless, this study could support the role of genetic predisposition in relation to the global resilience-vulnerability response to the COVID-19 pandemic.

## Conclusion

The present study showed that a lower PGS for the well-being spectrum and a higher PGS for neuroticism are associated with more vulnerable mental health profiles in response to the recurring stressors associated with the COVID-19 pandemic in Germany in 2020 and 2021. Despite the overall low predictive power of common genetic markers, we may conclude that methodological advances in GWAS based on latent phenotypes present promising tools to increase explained phenotypic variation when the mental health outcome and genetic predictors match well.

## Supplementary Information

Below is the link to the electronic supplementary material.

Online Resource 1: CovSocial study recruitment and participant exclusion criteria (DOCX 954 KB)

Online Resource 2: CovSocial study design, questionnaire items, and generation of the resilience-vulnerability trajectories (DOCX 224 KB)

Online Resource 3: Sample comparison and polygenic risk score correlations, validity, and specificity (XLSX 609 KB)

## Data Availability

Data will be made available upon request.
