# Supplementary material for: Genetic predisposition for negative affect predicts mental health burden during the COVID-19 pandemic
Source: Eur Arch Psychiatry Clin Neurosci. 2024 Apr 8;275(1):61–73. doi: 10.1007/s00406-024-01795-y (PMC11799032; doi:10.1007/s00406-024-01795-y)
Supplement: Supplementary file 1 — Online Resource 1: CovSocial study recruitment and participant exclusion criteria (DOCX 954 KB) [file 406_2024_1795_MOESM1_ESM.docx]

# Genetic Predisposition for Negative Affect Predicts Mental Health Burden during the Covid-19 Pandemic

Alicia M. Schowe*^1,2^, Malvika Godara*^3^, Darina Czamara^1^, Mazda Adli^4,5^, Tania Singer^3^ **†**, Elisabeth B. Binder^1^†

^1^Dept. Genes and Environment, Max Planck Institute of Psychiatry, Munich, Germany

^2^Graduate School of Systemic Neuroscience, Ludwig Maximilian University, Munich Germany

^3^Social Neuroscience Lab, Max Planck Society, 10557 Berlin, Germany; singer@social.mpg.de (T.S.)

^4^Charité Universitätsmedizin Berlin, Berlin, Germany

^5^Fliedner Klinik Berlin, Berlin, Center for Psychiatry, Psychotherapy and Psychosomatic Medicine, Germany.

* Correspondence: [alicia_schowe@psych.mpg.de](mailto:alicia_schowe@psych.mpg.de), malvika.godara@gv.mpg.de

† These authors contributed equally to this work.

**Recruitment, Dropouts, and Outlier Detection**

Participants were recruited during the period August 2020 to November 2020. Participants registered for the study through the CovSocial project webpage (www.covsocial.de) by making a personal account on the website (please Figure 1 for the dedicated landing page of the website/webapp in German and in English).


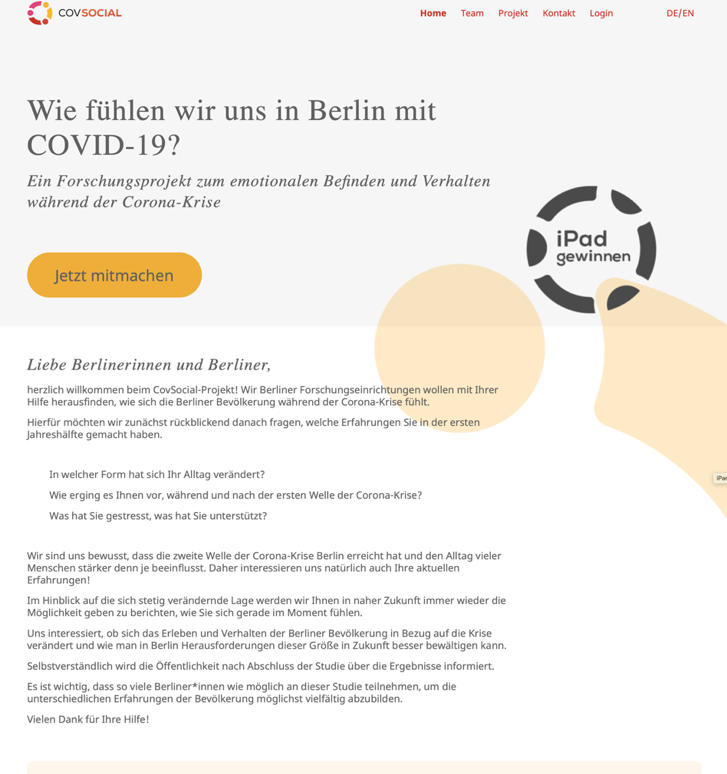

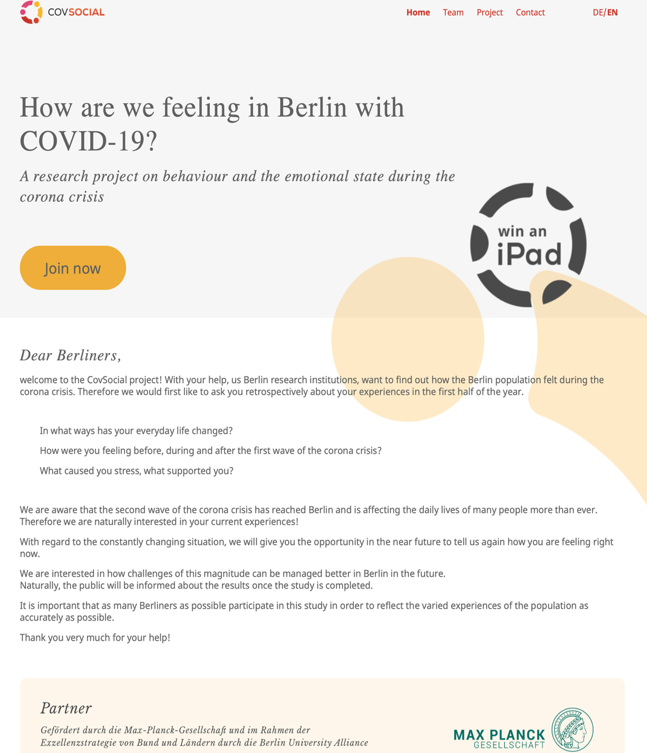


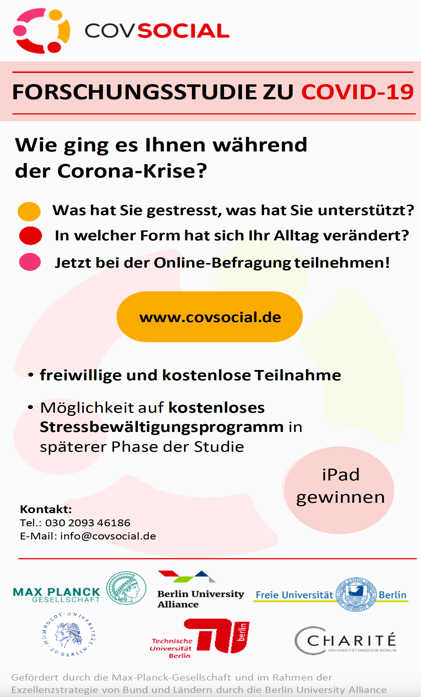

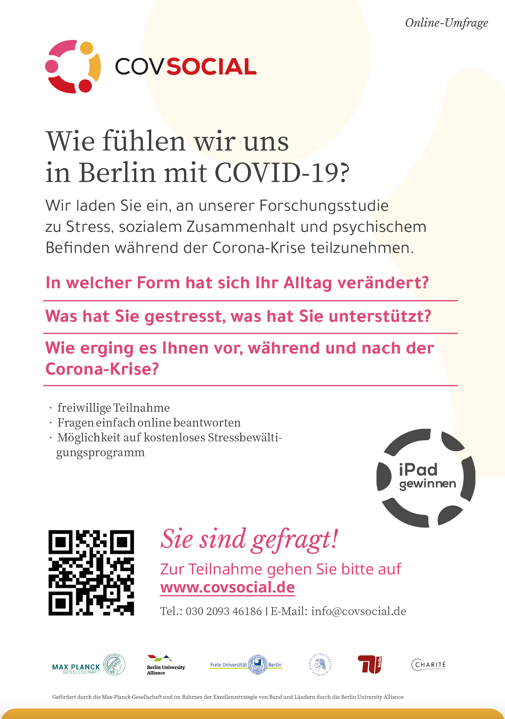

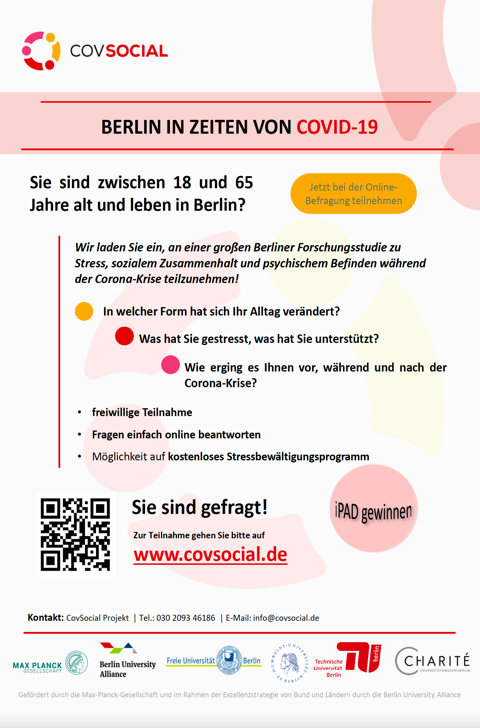


***Figure 1****. The landing page of the CovSocial website in German and English (Top panels).*

*Three different versions of the recruitment poster used in newspapers, posted on social media, and used as flyers that were put up in subway stations and public transport hubs (Bottom panels).*

**Recruitment procedure**

Participants were recruited from the population of the city of Berlin, Germany. For phase 1 of the CovSocial project, we aimed to recruit a total of 2000 participants between ages of 18 and 65 years old. The main inclusion criteria involved being able to understand the German language, and to be registered as a resident of the city of Berlin at the time of the assessment. Participants were recruited through the following variety of methods:

a) Randomly sampling 56,000 individuals through the Berlin registration office (Landesamt für Bürger- und Ordnungsangelegenheiten, Abt. II – Personalstands- und Einwohnerwesen, Referat Zentrale Einwohnerangelegenheiten (IIA), Friedrichstr. 219, 10969 Berlin). The residential addresses of the randomly chosen individuals were shared with the CovSocial research team by the registration office. Recruitment letters were sent to the addresses describing the study information, an appeal to participate, and a link to the study webpage/webapp,

b) Distribution of recruitment material via e-mail-lists of the academic and research institutions involved (Max Planck Society, Charité – Universitätsmedizin Berlin, and Humboldt University of Berlin),

c) Distribution of flyers at churches, and sports clubs,

d) Posting of recruitment material on social media,

e) Distribution of recruitment material in the form of advertisements in e-newspapers,

f) Posting flyers with recruitment material in the Berlin public transportation hubs and metro stops,

g) Additional recruitment using targeted chain-referral method wherein current participants are requested to refer the study recruitment to their social networks, friends, and family.

Figure 1 provides the original German version of the recruitment text that was used in the letters, flyers, posts, and advertisements. A total of 56,000 individuals were contacted for recruitment through the various methods mentioned above. 7214 individuals registered on the website to participate in the study. Figure 2 provides an overview of how many participants were recruited through the various avenues of recruitment.


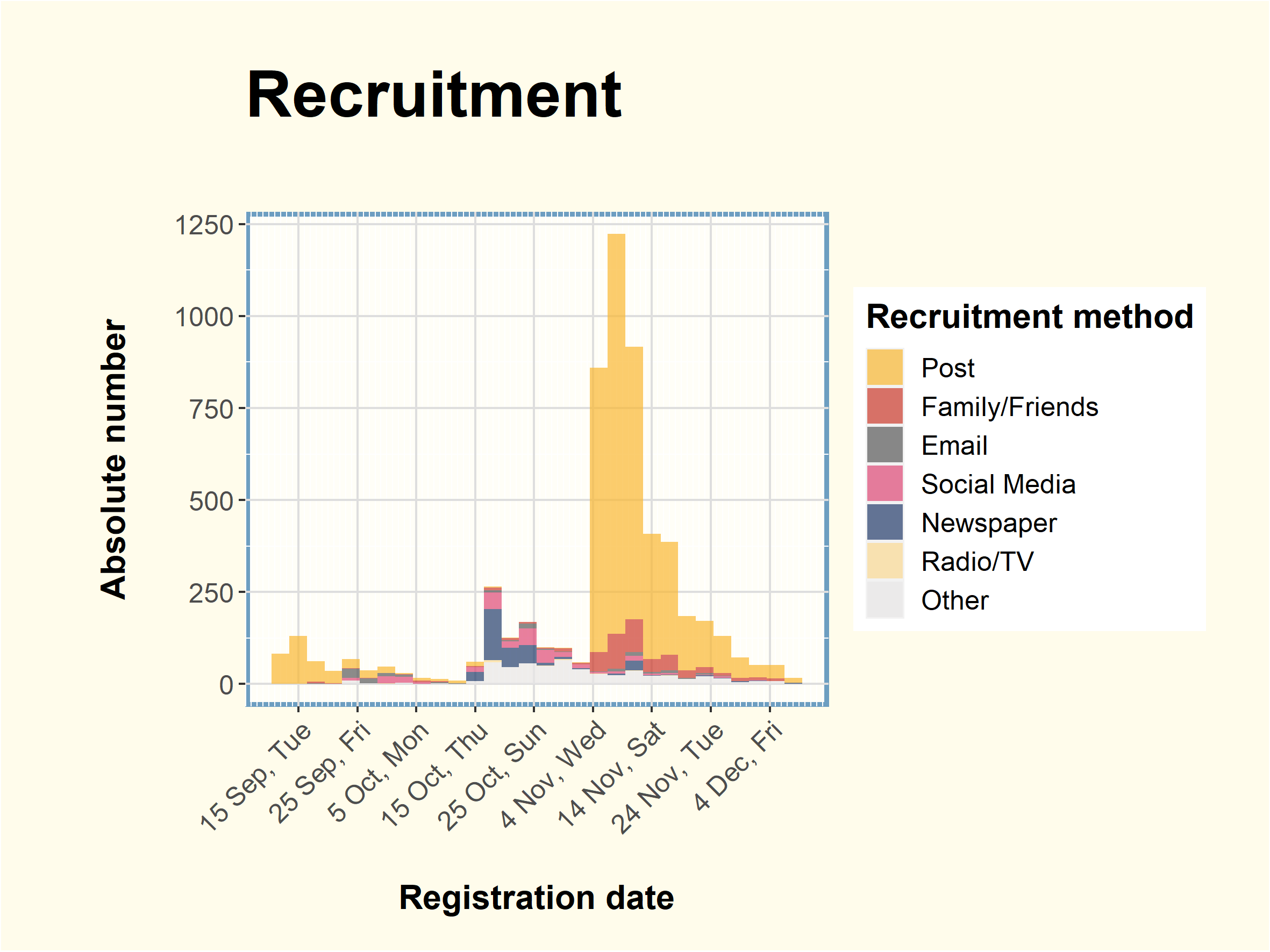


***Figure 2.*** *An overview of how many participants were recruited through the various avenues of recruitment over time.*

**Initial sample and dropouts**

The recruitment led to the registration of 7214 individuals to take part in the study. Out of this, only 5877 started and completed the first block of demographic questions. At every block, we saw further dropout in sample. Ultimately, 3681 individuals completed the entire survey, including demographic-related questionnaire, trait-level questionnaires, and state-level questionnaires for all three timepoints. The dropped-out participants either only registered to take part in the study or only provided responses to less than 7 blocks of questions, i.e., they did not complete all the questionnaires.

**Outlier detection**

In order to achieve our final sample for analysis, we first identified participants who met the exclusion criteria. The exclusion criteria were as follows: a) not being a resident of Berlin at the time of assessment, and b) being under 18 years of age or being over 65 years of age

If participants met any of the exclusion criteria, they were removed from further analyses. Based on the exclusion criteria, a total of 125 people were excluded for not living in Berlin (N = 44) and for being outside the age range (N = 81).

Next, in order to ensure the quality of self-report data provided by participants, several outlier checks were applied. First, a speed outlier check was applied based on how quickly participants completed a block of questionnaires. The speed was compared to a pre-defined threshold. The threshold was determined based on the speed of five young people, highly familiarized with nature of the study design and the content of questionnaires, clicking through the various blocks of questions. Out of the four, the fastest speed to click through the questions randomly was defined as the speed threshold for the blocks. If the participants completed a block of questionnaires with a speed that was below the defined threshold, they would get flagged in that block. Participants could collect as many as seven flags, one for each block of questions. All participants who had speed faster than the defined threshold on 2 blocks of questions were considered outliers and excluded from further analysis (N = 30).

A second form of outlier analysis was applied to validate the speed-based outliers, and this was termed as content-based outliers. This pertained to inconsistencies presented in the content of the answers to questions over the seven blocks. The following content-based inconsistencies were flagged:

1. Years of education < 8

2. Inconsistencies between degree of education (Abitur) and years of education (<11)

3. Inconsistencies between home office and office working hours (>120%)

4. Hours of work per week > 80

5. Inconsistencies between being a student (education) or not (job situation)

6. Inconsistencies between being a student and hours of work per week (>40)

The content-based flags were not used to remove outliers, but were used to check whether participants with speed flags also displayed at least one content-based inconsistency. Out of the 30 participants who were flagged for speed below threshold (in at least 2 blocks), 7 (i.e., 23%) were also flagged for content-based inconsistencies. Moreover, due to technical issues, a further three participants had missing data on blocks five (Trait 2) and seven (Trait 3), which consisted of the trait questions. Therefore, they were also excluded from further analysis. Lastly, one participant withdrew consent to use their data after the completion of the data assessment, and was therefore, removed from further analysis.
